# Loss of heterozygosity at 18q21 is indicative of recurrence and therefore poor prognosis in a subset of colorectal cancers

**DOI:** 10.1038/sj.bjc.6690144

**Published:** 1999-02

**Authors:** P Jernvall, M J Mäkinen, T J Karttunen, J Mäkelä, P Vihko

**Affiliations:** Biocenter Oulu and World Health Organization Collaborating Centre for Research on Reproductive Health;, University of Oulu, Kajaanintie 52 D, Oulu, FIN-90220, Finland; Department of Pathology, University of Oulu, Kajaanintie 52 D, Oulu, FIN-90220, Finland; Department of Surgery, University of Oulu, Kajaanintie 50, Oulu, FIN-90220, Finland; Department of Biosciences, Division of Biochemistry, University of Helsinki, Helsinki, FIN-00014, Finland

**Keywords:** chromosome 18, colorectal cancer, loss of heterozygosity, prognosis

## Abstract

Adjuvant therapies are increasingly used in colorectal cancers for the prevention of recurrence. These therapies have side-effects and should, thus, be used only if really beneficial. However, the development of recurrence cannot be predicted reliably at the moment of diagnosis, and targeting of adjuvant therapies is thus based only on the primary stage of the cancer. Loss of heterozygosity (LOH) in the long arm of chromosome 18 is suggested to be related to poor survival and possibly to the development of metastases. We studied the value of LOH at 18q21 as a marker of colorectal cancer prognosis, association with clinicopathological variables, tumour recurrence and survival of the patients. Of the 255 patients studied, 195 were informative as regards LOH status when analysed in primary colorectal cancer specimens using the polymerase chain reaction (PCR) and fragment analysis. LOH at 18q21 was significantly associated with the development of recurrence (*P*= 0.01) and indicated poor survival in patients of Dukes' classes B and C, in which most recurrences (82%) occurred. An increased rate of tumour recurrence is the reason for poor survival among patients with LOH at 18q21 in primary cancer. These patients are a possible target group for recurrence-preventing adjuvant therapies. © 1999 Cancer Research Campaign


British Journal of Cancer (1999) 79(5/6), 903–908
© 1999 Cancer Research Campaign
Article no. bjoc.1998.0144

### Loss of heterozygosity at 18q21 is indicative of recurrence and therefore poor prognosis in a subset of colorectal cancers

**P Jernvall[1], MJ Mäkinen[2], TJ Karttunen[2], J Mäkelä[3] and P Vihko[1,4]**

[1]Biocenter Oulu and World Health Organization Collaborating Centre for Research on Reproductive Health; [2]Department of Pathology, University of Oulu, Kajaanintie 52 D, FIN-90220 Oulu, Finland; [3]Department of Surgery, University of Oulu, Kajaanintie 50, FIN-90220 Oulu, Finland; [4]Department of Biosciences, Division of Biochemistry, FIN-00014 University of Helsinki, Finland

**Summary** Adjuvant therapies are increasingly used in colorectal cancers for the prevention of recurrence. These therapies have side-effects and should, thus, be used only if really beneficial. However, the development of recurrence cannot be predicted reliably at the moment of diagnosis, and targeting of adjuvant therapies is thus based only on the primary stage of the cancer. Loss of heterozygosity (LOH) in the long arm of chromosome 18 is suggested to be related to poor survival and possibly to the development of metastases. We studied the value of LOH at 18q21 as a marker of colorectal cancer prognosis, association with clinicopathological variables, tumour recurrence and survival of the patients. Of the 255 patients studied, 195 were informative as regards LOH status when analysed in primary colorectal cancer specimens using the polymerase chain reaction (PCR) and fragment analysis. LOH at 18q21 was significantly associated with the development of recurrence ($P$ = 0.01) and indicated poor survival in patients of Dukes' classes B and C, in which most recurrences (82%) occurred. An increased rate of tumour recurrence is the reason for poor survival among patients with LOH at 18q21 in primary cancer. These patients are a possible target group for recurrence-preventing adjuvant therapies.

**Keywords:** chromosome 18; colorectal cancer; loss of heterozygosity; prognosis

Colorectal cancer is the second most common cancer in many Western countries. Outcome among such patients is often poor, with a mean 5-year relative survival rate of around 50% (Mäkelä et al, 1995; Sant et al, 1995). Prognosis is dependent on the stage of the disease at the moment of diagnosis. In patients with the least advanced Dukes' classes A and B tumours, 5-year cumulative survival is as good, 95% and 70% respectively, whereas it is around 30% in patients with the more advanced Dukes' class C, and in patients with the primarily metastatic Dukes' class D it is only 2% (Arveux et al, 1997).

Surgery is the most efficient therapy in colorectal cancer. Despite radical surgical treatment, tumour recurrence occurs in 30–40% of cases (Mäkelä et al, 1995; Obrand and Gordon, 1997). The use of adjuvant therapies in preventing recurrence, especially in patients with Dukes' class C colon and rectal cancers, and also in some patients with Dukes' class B rectal cancer, is continuously increasing. These therapies have side-effects, and thus they should be given only to those who really benefit from them, which actually means they should be given to those patients prone to develop a recurrent tumour (Fielding et al, 1992; Swedish Rectal Cancer Trial, 1996). These recurrences cannot be reliably predicted by any means at the moment of primary operation. Thus, targeting of adjuvant therapies is nowadays based only on the primary stage of the disease, not on the presence of any specific 'marker' related to the development of recurrent tumours (Vernava et al, 1994).

Allelic loss (loss of heterozygosity, LOH) in the long arm of chromosome 18 can be detected in about 60–70% of colorectal cancer cases. The most frequent area of loss is 18q21–qter, where the candidate tumour-suppressor genes *MADR2*, *DPC4* and *DCC* are known to reside (Fearon et al, 1990; Thiagalingam et al, 1996). The area has not been fully examined, and it is believed that other tumour-suppressor genes are likely to be found there in the near future (Eppert et al, 1996). In previous studies, mostly carried out with relatively small or selected patient groups, LOH in this area has been suggested to be predictive of metastasis and poor prognosis (O'Connell et al, 1992; Iino et al, 1994; Jen et al, 1994; Frank et al, 1997).

In order to clarify the value of LOH at 18q21 as a marker in colorectal cancer prognosis, we have undertaken a comprehensive analysis of 255 (of which 195 were informative) colorectal cancers as regards LOH at 18q21 and correlated our findings with patient outcome. Allele status was correlated with clinical and pathological features of the tumour, such as its location, stage of disease, histological grade, mucinous cancer type and development of recurrence. Survival analysis was carried out in correlation with LOH status.

### MATERIALS AND METHODS

### Patients

The status of chromosome 18 long arm area 21 was studied in 255 patients operated upon in 1986–91 and 1993–96 for primary colorectal cancer in order to evaluate the possible clinical importance of loss of heterozygosity in this region. Of these 255

*Received 24 February 1998*
*Revised 26 May 1998*
*Accepted 8 July 1998*

*Correspondence to:* P Vihko, Biocenter Oulu and World Health Organization Collaborating Centre for Research on Reproductive Health, University of Oulu, Kajaanintie 50, FIN-90220 Oulu, Finland patients, 195 were informative as regards LOH status. Excluded from the study were 28 replication error-positive cases and 32 cases that were uninformative as a result of homozygous genotype or sampling problems. In data handling, only cases informative as regards LOH were taken into account.

Of the 195 patients, 105 (54%) were men and 90 (46%) were women. The age distributions were from 24 to 88 years in men (mean 66) and from 38 to 93 years in women (mean 68). Follow-up was organized as described by Mäkelä et al (1995). The follow-up time ranged from 2 to 138 months (mean 40) and ended on the 30 August 1997 or at the moment of death. Case records and cancer registry files (Finnish Cancer Registry) were used to evaluate the medical history of the patients and clinical aspects of the disease. The original microscopy slides were reviewed separately by two pathologists to confirm the pathological grading and staging. Grading was carried out according to the World Health Organization histological classification system (Jass and Sobin, 1989). Patients were in histological grades as follows: I, 46 (26%); II, 107 (61%); III, 23 (13%). Of the tumours, 19 were of mucinous type. Staging was carried out on the basis of histological and clinical examinations according to the Turnbull modification of Dukes' classification: A, 48 (24.6%); B, 78 (40.0%); C, 45 (23.1%); and D, 24 (12.3%) (Turnbull et al, 1967). Tumours occurring from the caecum to the splenic flexure were considered proximal (49, 25%), and those from the descending colon to the rectum distal (146, 75%) (Ponz de Leon et al, 1992).

In the analyses related to recurrence, only patients curatively operated upon prior to 30 August 1995 were included, in order to achieve sufficient follow-up time. A minimum follow-up time of 2 years was established because the majority, about 85%, of colorectal cancer recurrence is known to occur within 2 years of the primary operation (Mäkelä et al, 1995). Recurrence is considered local if in the area of anastomosis or having invaded out of the primary resection area but having no distant metastases, and distant if there is distant metastasis (Mäkelä et al, 1995).

**Table 1** LOH in relation to clinicopathological features of the tumours

| | LOH– | | LOH+ | | *P*-value |
|---|---|---|---|---|---|
| | *n* | % | *n* | % | |
| Dukes' class | | | | | |
| A | 31 | 65 | 17 | 35 | 0.028 |
| B | 34 | 44 | 44 | 56 | |
| C | 16 | 36 | 29 | 64 | |
| D | 13 | 54 | 11 | 46 | |
| Grade | | | | | |
| I | 25 | 54 | 21 | 46 | 0.19 |
| II | 44 | 41 | 63 | 59 | |
| III | 13 | 57 | 10 | 43 | |
| Mucinotic | 12 | 63 | 7 | 37 | 0.16 |
| Non-mucinotic | 82 | 47 | 94 | 53 | |
| Tumour location | | | | | |
| Caecum | 13 | 65 | 7 | 35 | 0.13 |
| Ascending colon | 7 | 70 | 3 | 30 | |
| Hepatic flexure | 2 | 33 | 4 | 66 | |
| Transverse colon | 3 | 43 | 4 | 57 | |
| Splenic flexure | 3 | 50 | 3 | 50 | |
| Descending colon | 5 | 71 | 2 | 29 | |
| Sigmoid colon | 15 | 34 | 29 | 66 | |
| Recto-sigmoid | 6 | 75 | 2 | 25 | |
| Rectum | 40 | 46 | 47 | 54 | |

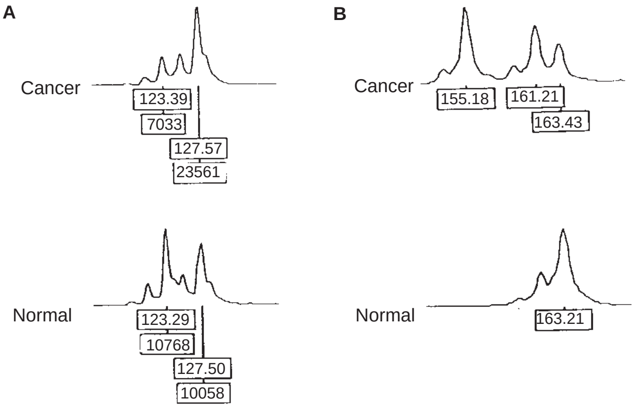

**Figure 1** (**A**) A cancer representative of allelic loss (upper) and a corresponding normal tissue sample (lower). (**B**) A cancer representative of RER-positivity (upper) and a corresponding normal tissue sample (lower)

### DNA extraction

Blood samples and fresh tissue specimens were collected at the primary operation from those patients operated upon in 1993–96. Paraffin-embedded archival material was available from those patients primarily operated upon in 1986–91.

The specimens were evaluated by two experienced pathologists as described previously (Jernvall et al, 1997). Areas with the highest proportion of neoplastic cells were dissected and used as tumour samples. Normal tissue or blood was used as a control. DNA extraction from fresh tissue and blood samples was performed as previously described (Elo et al, 1995), as was DNA extraction from paraffin-embedded tissue (Wright and Manos, 1990).

### Microsatellite markers and polymerase chain reaction (PCR)

Microsatellite markers were used to study the status of chromosome 18 long arm region 21. Cases with a minimum of two informative microsatellite markers in this region, were included in the study. A tumour expressing LOH in a minimum of two markers was considered LOH positive. Those samples expressing new alleles were considered replication error positive (RER positive) and were excluded from the study (Jen et al, 1994). The markers, chosen from the Gènèthon human genetic linkage map (ftp://ftp.resgen.com/pub), were, from telomere to centromere, D18S58, D18S61, D18S55, D18S69, DCC, D18S474 and D18S65 (Dib et al, 1996). In each primer pair one primer was fluorescently labelled for allele detection using a 381 A DNA Synthesizer (Applied Biosystems, Foster City, CA, USA). The PCR reactions contained 100 ng of DNA as template, 0.6 μg of each primer, four deoxynucleotidetriphosphates (200 μM of each; Pharmacia LKB Biotechnology, Uppsala, Sweden), 2 μl of 10 × reaction buffer IV (200 mM ammonium sulphate, 750 mM Tris-HCl, pH 9.0 at 25°C, 0.1% w/v Tween), 1.75 mM magnesium chloride and 0.5 U of Red Hot DNA polymerase (Advanced Biotechnologies, Surrey, UK) in a 20 μl reaction volume. The PCRs were run in the following conditions: denaturation at 95°C for 3 min followed by 35 cycles (fresh tissue and blood) or 45 cycles (paraffin-embedded samples)

 *© Cancer Research Campaign 1999*

**Table 2** Recurrence in the LOH-positive and LOH-negative groups in relation to Dukes' class and tumour location

| | Recurrence | | | | | |
|---|---|---|---|---|---|---|
| | Overall rate | | LOH+ | | LOH– | |
| | *n* | % | *n* | % | *n* | % |
| Dukes' class | | | | | | |
| A | 6/32 | 19 | 2 | 33 | 4 | 67 |
| B | 15/59 | 25 | 12 | 80 | 3 | 20 |
| C | 18/32 | 56 | 12 | 67 | 6 | 33 |
| D | 1/2 | 50 | 1 | 100 | 0 | 0 |
| Location | | | | | | |
| Proximal | 5/26 | 19 | 3 | 60 | 2 | 40 |
| Distal | 35/99 | 35 | 24 | 69 | 11 | 31 |

consisting of denaturation at 95°C for 1 min, annealing at 53°C for 0.45–1.5 min and extension at 72°C for 1 min. Final extension after the cycles was at 72°C for 10 min.

### Gel analysis

The amplification product of each marker was mixed with 0.25 µl of GS500-TAMRA size standard and 3.25 µl of loading buffer (Applied Biosystems). From the mixture, 1.5 µl was then loaded onto a Long Ranger 5% gel (FMC BioProducts, Rockland, ME, USA) and the fragments were separated using an ABI Prism 377 automated fluorescent sequencer and further analysed with GeneScan and Genotyper softwares (Applied Biosystems). The two alleles of each sample were assigned according to the two peaks of greatest height. Peak areas were compared with those from the corresponding normal samples and an allele ratio was calculated as described previously (Cawkwell et al, 1993). Those samples with allele ratios <0.6 or >1.66 were considered LOH positive. An example representative of allelic loss is shown in Figure 1a, and an example representative of RER positivity is shown in Figure 1b.

### Statistical analysis

Pearson's $\chi^2$ test or exact tests (when appropriate) were used to analyse statistical correlations between LOH and other variables. Cumulative survival was assessed by the Kaplan–Meier method and analysed by log-rank analysis. Perioperative deaths and other deaths unrelated to colorectal carcinoma were censored at the time of occurrence of the survival analysis. Multivariate analysis was carried out by Cox regression analysis to determine independent factors affecting survival. In all analyses, $P < 0.05$ was considered statistically significant. The statistical procedures were performed using SPSS software (SPSS, Chicago, IL, USA).

### RESULTS

Of the 255 patients, 195 were informative as regards LOH status. Loss of heterozygosity at 18q21 was found in 101 cases (52%). It was equally common in women 47/90 (52%) and in men 54/105 (51%). The majority of instances of loss were found in tumours of the distal colon or rectum: 80 of 101 LOH-positive tumours (79%). LOH was most frequently found in tumours of Dukes'

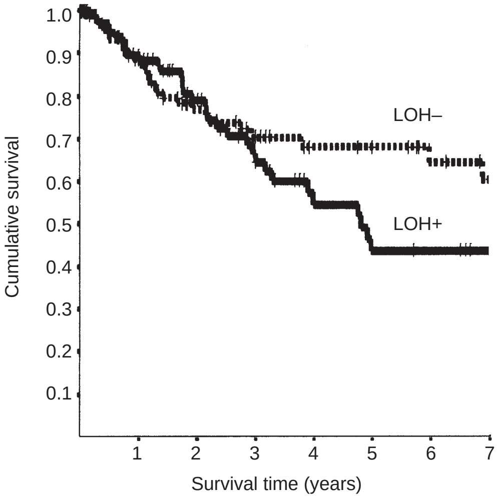

**Figure 2** Cumulative 5-year survival rates of LOH-positive (*n* = 101) and LOH-negative (*n* = 94) patients classes B and C. In the primarily metastatic Dukes' D class, LOH was seen in less than half of the cases, and in the least advanced Dukes' class A in only one third of the cases. LOH was significantly associated ($P = 0.028$) with the primary stage of the disease, but it was not associated with the primary metastasis site in Dukes' D tumours. Nor was it associated with the histological grade or size of the tumour. Nineteen mucinous tumours were identified, seven of them being LOH positive. Although LOH was less frequently found in mucinous tumours, LOH negativity was not significantly associated with the mucinous phenotype of cancer. Clinicopathological features such as Dukes' class, histological grade, mucinocity and tumour location in relation to LOH status are shown in Table 1. Analyses related to tumour recurrence were performed on data from a group of 125 patients that had been curatively operated upon at least 2 years prior to the end point of this study in order to achieve sufficient follow-up time. Tumour recurrence was detected in 32% (40) of the cases; of these 57% (23) were local and 43% (17) were distant recurrences. Of the recurrences, 85% occurred during the first 2 years of follow-up. The appearance of a recurrent tumour was significantly associated with LOH at 18q21 ($P = 0.01$). Of the primary tumours that recurred, 68% were LOH positive. The absence of LOH indicated recurrence-free disease in 78% of the cases ($P = 0.01$). Most recurrences appeared in patients with primary tumours of Dukes' classes B and C (82%) ($P = 0.005$). Of the class B tumours, 25% recurred and, of the class C tumours, 56% recurred. Of the recurrences, 87% (35) appeared in patients with primary disease of the distal colon or rectum. The rate of recurrence in the LOH-positive and LOH-negative groups in relation to Dukes' class and location of the tumour is shown in Table 2. The time from primary operation to diagnosis of recurrence did not differ between the LOH-positive and the LOH-negative group; nor was the site of recurrence associated with LOH status. Cumulative survival since detection of recurrent disease was 62% at 1 year, 29% at 2 years and 11% at 3 years, being similar in both groups.

Multivariate analysis was performed in order to identify independent factors affecting survival. Dukes' class, grade, tumour location, age, gender and LOH status were included in the analysis as covariates. Three separate analyses were carried out. In phase I,

© Cancer Research Campaign 1999

**Table 3** Three-phase multivariate analysis for identification of prognostic factors for colorectal cancer. Only those covariates that were statistically significant in the analysis are presented. Phase I, all patients; phase II, patients included in the recurrence analysis; phase III, Dukes' class B patients that were included in the recurrence analysis

| Covariate | Relative hazard | 95% CI | *P*-value | Phase |
|---|---|---|---|---|
| Grade | | | | |
| I (ref) | | | | I |
| II | 1.3 | 0.6–2.7 | NS | |
| III | 3.2 | 1.2–8.2 | 0.01 | |
| Location (prox-dist) | 1.9 | 0.9–3.9 | 0.05 | |
| Dukes' | | | | |
| A (ref) | | | | |
| B | 2.1 | 0.7–5.8 | NS | |
| C | 4.2 | 1.5–11.7 | 0.005 | |
| D | 30 | 9.7–93 | <0.001 | |
| Dukes' | | | | II |
| A (ref) | | | | |
| B | 2.0 | 0.67–6.0 | NS | |
| C | 5.0 | 1.6–15.3 | 0.004 | |
| D | 39 | 3.9–388 | 0.001 | |
| LOH | 3.9 | 0.9–9.1 | 0.04 | III |

NS, not significant.

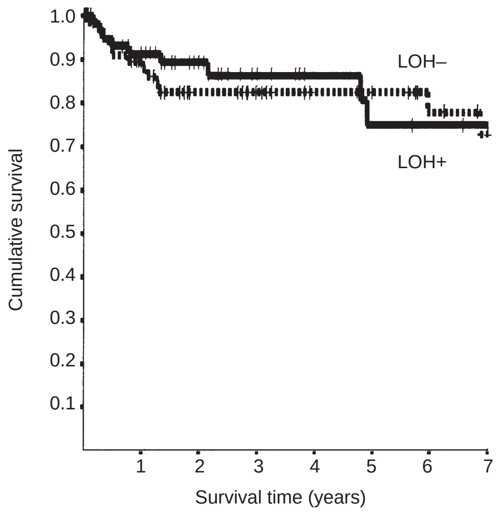

**Figure 3** Cumulative 5-year survival rates of LOH-positive ($n$ = 63) and LOH-negative ($n$ = 73) patients with no tumour recurrence all 195 patients were included in the analysis, in phase II only those patients included in the recurrence analysis were involved and in phase III the analysis was carried out separately in each Dukes' class. The results of the analyses are shown in Table 3.

The overall cumulative 5-year survival rate for LOH-negative and LOH-positive patients was 67% and 46% respectively (Figure 2). When Dukes' D cases were excluded from the analysis, the 5-year cumulative survival rates for LOH-negative and LOH-positive cases was 75% and 50% respectively. When the grade III and Dukes' D tumours were both excluded from the survival analysis, as these appear to be strong, independent factors affecting survival, the role of LOH at 18q21 became even more obvious, with a 5-year cumulative survival rate of 78% in the LOH-negative group compared with 50% in the LOH-positive group ($P$ = 0.06). Survival analysis was accordingly carried out in the group of patients included in the recurrence analysis. In this group, the result was very similar to that seen with the exclusion of Dukes' D and grade III patients. The 5-year cumulative survival rates of LOH-positive patients (48%) and LOH-negative patients (79%) differed significantly ($P$ = 0.05).

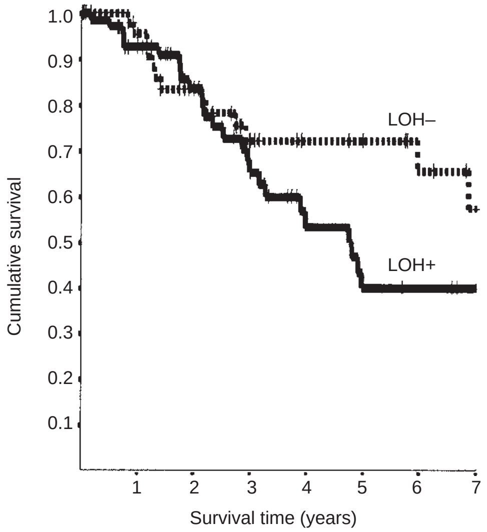

**Figure 4** Cumulative 5-year survival rates of LOH-positive ($n$ = 72) and LOH-negative ($n$ = 50) patients of Dukes' classes B and C

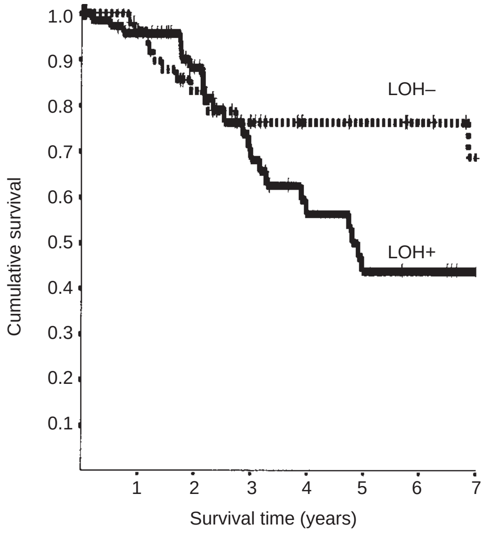

**Figure 5** Cumulative 5-year survival rates of LOH-positive ($n$ = 80) and LOH-negative ($n$ = 66) patients with distally located tumours

In all these survival analyses, a marked difference in the survival rates of LOH-positive and LOH-negative cases only became apparent after a 3-year period. The effect of recurrence on survival was studied by exclusion of all recurrent cases in the survival analysis. The survival rates of LOH-positive and LOH-negative patients were then similar to each other (Figure 3).

LOH positivity was related to poor survival in Dukes' classes B and C, as shown in Figure 4. In class B, the 5-year survival rate of LOH-negative patients was 79%, compared with 57% in LOH-positive patients. In Dukes' class C, the corresponding percentages were 44% and 21%. LOH positivity was not correlated to survival

 *© Cancer Research Campaign 1999*

in Dukes' classes A and D.

The association between LOH and survival was further analysed in relation to location of the primary tumour. As regards distal tumours, the 5-year survival rate was 76% for LOH-negative patients and 47% for LOH-positive patients (Figure 5). Patients with proximal LOH-positive tumours had a survival rate only slightly worse than that associated with LOH-negative ones.

### DISCUSSION

Loss of heterozygosity at 18q21 in primary colorectal cancers was studied in order to clarify its possible role as a marker of disease outcome. Of the 195 informative cases, 52% were LOH positive, a percentage somewhat lower than that reported in other studies. Because of the very careful selection of sample tissues by two experienced pathologists, it is unlikely that contamination by normal tissue would have affected the result. Additionally, LOH was most common in diseases of Dukes' classes B and C, with frequencies of LOH positivity of 56% and 64% respectively, similar to those reported previously (60–67%) (O'Connell et al, 1992; Jen et al, 1994).

Our results show that LOH at 18q21 is correlated significantly with the appearance of recurrent disease ($P$ = 0.01). They also show that LOH is associated with poor survival, and that this is due to an increased rate of recurrence in LOH-positive tumours. Of the primary tumours that recurred, 68% were LOH positive. Additionally, LOH negativity indicated recurrence-free disease in 78% of the cases. LOH positivity and disease recurrence were most commonly seen in patients belonging to Dukes' classes B and C. The higher rate of LOH positivity and recurrences seen in distal compared with proximal cases may be related to the fact that distal cancers were more often of Dukes' classes B and C than the proximal ones (data not shown). In survival analysis, those with LOH-positive disease had a survival rate similar to that of LOH-negative cases for the first 3 years. After that, the survival rate of LOH-positive patients sharply decreased by 20%–27% compared with that of LOH-negative patients (Figures 2, 4 and 5), resulting in a marked difference in the survival rates of LOH-positive and LOH-negative patients, which, however, as a result of limitations in the survival analysis used, appeared statistically significant in only one of the analyses. This decrease in the survival rate was clearly associated with a higher rate of recurrence and subsequently an increased rate of cancer-related death in LOH-positive cases compared with LOH-negative ones. The 3-year period of similar survival rate of these two groups is the time during which these recurrences develop (85% in 2 years) and patients with recurrent disease begin to lose the fight against cancer and die (the 2-year survival rate after recurrence was 29%).

Additional analysis revealed that LOH positivity was significantly correlated with poor survival in Dukes' class B, and it also indicated poor survival in class C, both being classes in which most cases of recurrence developed (82%; Figure 4). The correlation between LOH positivity and poor survival was markedly clearer in distal than in proximal cancers, 87% of all recurrences being in distal cancers (Figure 5). The difference seen in the survival rates of LOH-positive and LOH-negative cases disappeared after exclusion of all the recurrent cases (Figure 3). Hence, an increased rate of recurrence appears to be the reason for poor survival among patients with LOH at 18q21 in primary tumours in Dukes' classes B and C.

The value of LOH at 18q as a prognostic indicator has been assessed previously in a study by O'Connell et al (1992). They suggested a possible correlation between LOH at 18q in Dukes' B and C tumours and poor survival. They also noticed an increased rate of recurrence in LOH-positive cases, but the results were not statistically significant because they had only 90 patients in the study. Jen et al (1994) pointed out the importance of LOH in this region in stage II tumours, with a survival rate of 54% in LOH-positive patients compared with 93% in LOH-negative ones. However, they found no such correlation as regards stage III disease, and their study was not informative regarding the association between LOH positivity and the rate of recurrent disease.

We detected LOH at 18q21 most commonly in Dukes' B and C tumours, a result similar to that of previous studies (Kikuchi-Yanoshita et al, 1992; Iino et al, 1994). The rate of LOH in cases of Dukes' class D was somewhat lower than in cases of classes B and C. This is contradictory, because genetic changes are known to accumulate as cancer advances (Iino et al, 1994; Jen et al, 1994). The LOH-positive tumours tended to be located more often in the distal colon or rectum, as reported previously (Jen et al, 1994). The correlation with mucinous tumour type was also similar to that previously reported (Hedrick et al, 1994). The proportions of LOH-positive and LOH-negative patients in the three histological grades differed from those previously reported, apparently as a consequence of different patient populations used in these studies. Jen et al (1994) studied patients with disease stages II and III only, whereas our patients were not selected according to the primary stage of the disease. Variation in determination of histological grade can also occur, depending on the person carrying out the grading (Jen et al, 1994). We saw no correlation between the appearance of LOH and the site of primary metastasis. Kato et al (1996) found a correlation between LOH at 18q and liver metastasis. However, they studied primarily metastatic and recurrent tumours all as one group.

We conclude that LOH at 18q21 is a very important feature of colorectal cancer progression, as our results suggest a central role for it in recurrence development and thus in poor prognosis. Examination of LOH at 18q21 in primary colorectal cancers of Dukes' classes B and C can help to find patients that are prone to recurrence and consequently need intensified adjuvant therapy and more careful follow-up.

### ACKNOWLEDGEMENTS

This work was supported by the Finnish Cancer Foundation. We thank Ms A Kujari for her technical assistance and Dr R Bloigu for advice on statistical analysis.

### REFERENCES

Arveux I, Boutron MC, Arveux P, Liabeuf A, Pfitzenmeyer P and Faivre J (1997) Colon cancer in the elderly: evidence for major improvements in health care and survival. *Br J Cancer* **76**: 963–967

Cawkwell L, Bell SM, Lewis FA, Dixon MF, Taylor GR and Quirke P (1993) Rapid detection of allele loss in colorectal tumours using microsatellites and fluorescent DNA technology. *Br J Cancer* **87**: 1262–1267

Dib C, Faure S, Fizames C, Samson D, Drouot N, Vignal A, Millasseau P, Marc S, Hazan J, Seboun E, Lathrop M, Gyapay G, Morissette J and Weissenbach J (1996) A comprehensive genetic map of the human genome based on 5,264 microsatellites. *Nature* **380**: 152–154

Elo JP, Kvist L, Leinonen K, Isomaa V, Henttu P, Lukkarinen O and Vihko P (1995) Mutated human androgen-receptor gene detected in a prostatic-cancer patient is also activated by estradiol. *J Clin Endocrinol Metab* **80**: 3494–3500

Eppert K, Scherer SW, Ozcelik H, Pirone R, Hoodless P, Kim H, Tsui L-C, Bapat B,

© Cancer Research Campaign 1999 

Gallinger S, Andrulis IL, Thomsen GH, Wrana JL and Attisano L (1996) MADR2 maps to 18q21 and encodes a TGFβ-regulated MAD-related protein that is functionally mutated in colorectal carcinoma. *Cell* **86**: 543–552
Fearon ER, Cho KR, Nigro JM, Kern SE, Simons JW, Ruppert JM, Hamilton SR, Preisinger AC, Thomas G, Kinzler KW and Vogelstein B (1990) Identification of a chromosome 18q gene that is altered in colorectal cancers. *Science* **247**: 49–56
Fielding LP, Hittinger R, Grace RH and Fry JS (1992) Randomised controlled trial of adjuvant chemotherapy by portal-vein perfusion after curative resection for colorectal adenocarcinoma. *Lancet* **340**: 502–506
Frank CJ, McClatchey KD, Devaney KO and Carey TE (1997) Evidence that loss of chromosome 18q is associated with tumor progression. *Cancer Res* **57**: 824–827
Hedrick L, Cho KR, Fearon ER, Wu T-C, Kinzler KW and Vogelstein B (1994) The DCC gene product in cellular differentiation and colorectal tumorigenesis. *Genes Dev* **8**: 1174–1183
Iino H, Fukayama M, Maeda Y, Koike M, Mori T, Takahashi T, Kikuchi-Yanoshita R, Miyaki M, Mizuno S and Watanabe S (1994) Molecular genetics for clinical management of colorectal carcinoma. 17p, 18q, and 22q loss of heterozygosity and decreased DCC expression are correlated with the metastatic potential. *Cancer* **73**: 1324–1331
Jass JR and Sobin LH (1989) Histological typing of intestinal tumors. In *World Health Organization*. *International Classification of Tumors*, 2nd edn: Springer: Berlin
Jen J, Kim H, Piantadosi S, Liu Z-F, Levitt RC, Sistonen P, Kinzler KW, Vogelstein B and Hamilton SR (1994) Allelic loss of chromosome 18q and prognosis in colorectal cancer. *N Engl J Med* **331**: 213–221
Jernvall P, Mäkinen M, Karttunen T, Mäkelä J and Vihko P (1997) Conserved area mutations of the p53 gene are concentrated in distal colorectal cancers. *Int J Cancer (Pred Oncol*) **74**: 97–101
Kato M, Ito Y, Kobayashi S and Isono K (1996) Detection of DCC and Ki-ras gene alterations in colorectal carcinoma tissue as prognostic markers for liver metastatic recurrence. *Cancer* **77**: 1729–1735
Kikuchi-Yanoshita R, Konishi M, Fukunari H, Tanaka K and Miyaki M (1992) Loss of expression of the DCC gene during progression of colorectal carcinomas in familial adenomatous polyposis and non-familial adenomatous polyposis patients. *Cancer Res* **52**: 3801–3803
Mäkelä JT, Laitinen ST and Kairaluoma MI (1995) Five-year follow-up after radical surgery for colorectal cancer. *Arch Surg* **130**: 1062–1067
Obrand DI and Gordon PH (1997) Incidence and patterns of recurrence following curative resection for colorectal carcinoma. *Dis Colon Rectum* **40**: 15–24
O'Connell MJ, Schaid DJ, Ganju V, Cunningham J, Kovach JS and Thibodeau SN (1992) Current status of adjuvant chemotherapy for colorectal cancer. Can molecular markers play a role in predicting prognosis? *Cancer* **70**: 1732–1739
Ponz de Leon M, Sant M, Micheli A, Sacchetti C, Digregorio C, Fante R, Zanghieri G, Melotti G and Gatta G (1992) Clinical and pathological prognostic indicators in colorectal cancer. *Cancer* **69**: 626–635
Sant M, Capocaccia R, Verdecchia A, Gatta G, Micheli A, Mariotto A, Hakulinen T, Berrino F and The Eurocare Working Group (1995) Comparisons of colon-cancer survival among European countries: The Eurocare Study. *Int J Cancer* **63**: 43–48
Swedish Rectal Cancer Trial (1996) Local recurrence rate in a randomised multicentre trial of preoperative radiotherapy compared with operation alone in resectable rectal carcinoma. *Eur J Surg* **162**: 397–402
Thiagalingam S, Lengauer C, Leach FS, Schutte M, Hahn SA, Overhauser J, Willson JKV, Markowich S, Hamilton SR, Kern SE, Kinzler KW and Vogelstein B (1996) Evaluation of candidate tumour suppressor genes on chromosome 18 in colorectal cancers. *Nature Genet* **13**: 343–346
Turnbull RB, Kyle K, Watson FR and Spratt J (1967) Cancer of colon: the influence of the no-touch isolation technic on survival rates. *Ann Surg* **166**: 420–427
Vernava AM, Longo WE, Virgo KS, Coplin MA, Wade TP and Johnson FE (1994) Current follow-up strategies after resection of colon cancer. Results of a survey of members of the American Society of Colon and Rectal Surgeons. *Dis Colon Rectum* **37**: 573–583
Wright DK and Manos MM (1990) Sample preparation from paraffin-embedded tissues. In *PCR Protocols*: *A Guide to Methods and Applications*, Innis MA, Gelfand DH, Sninsky JJ and White TJ (eds), pp. 153–158. Academic Press: San Diego

*© Cancer Research Campaign 1999*
